# Bacterial profile, antimicrobial susceptibility patterns, and associated factors of community-acquired pneumonia among adult patients in Gondar, Northwest Ethiopia: A cross-sectional study

**DOI:** 10.1371/journal.pone.0262956

**Published:** 2022-02-01

**Authors:** Muluneh Assefa, Abiye Tigabu, Teshome Belachew, Belay Tessema

**Affiliations:** Department of Medical Microbiology, School of Biomedical and Laboratory Sciences, College of Medicine and Health Sciences, University of Gondar, Gondar, Ethiopia; Suez Canal University, EGYPT

## Abstract

**Introduction:**

Community-acquired pneumonia is associated with higher morbidity, hospitalization, and mortality in adults. Likewise, antimicrobial resistance has increased in recent decades in Ethiopia. Therefore, this study was aimed to determine the bacterial isolates, their antimicrobial susceptibility patterns, and factors associated with community-acquired pneumonia among adult patients in Gondar, Northwest Ethiopia.

**Materials and methods:**

This institutional-based cross-sectional study was conducted from April to June 2021. Sociodemographic, clinical, and other relevant data were collected using a pre-tested questionnaire. A total of 312 sputum specimens were collected using sputum cups and inoculated into blood agar, chocolate agar, mannitol salt agar, and MacConkey agar plates, which were then incubated at 37°C for 24 hours. The bacterial isolates were identified based on Gram staining, colony characteristics, and biochemical tests. Antimicrobial susceptibility testing was performed using the Kirby-Bauer disk diffusion method. Inducible clindamycin resistance among the *S*. *aureus* isolates was detected by the D-test. Data were entered using EPI data version 4.6 and analyzed using SPSS version 20. P-value ≤ 0.05 at 95% CI was considered statistically significant.

**Results:**

Of 312 cases, 39.4% (n = 123; 95% CI: 34.1%–44.9%) were found to have culture-confirmed pneumonia. The most common isolates were *K*. *pneumoniae* (31.0%, n = 39), *S*. *pneumoniae* (26.2%, n = 33), and *S*. *aureus* (20.6%, n = 26). The gram-positive bacteria were susceptible to chloramphenicol (100%) and clindamycin (96.6%). Gram-negative bacteria were susceptible to gentamicin (87.5%), azithromycin (87.1%), ciprofloxacin (86.6%), and ceftriaxone (79.0%) but highly resistant to ampicillin (100%), followed by tetracycline (87.1%), doxycycline (86.4%), co-trimoxazole (80.6%), and amoxicillin-clavulanic acid (79.0%). Overall, 72.2% of the isolates were multi-drug resistant to *K*. *pneumoniae* (94.9%, n = 37), *E*. *coli* (93.8%, n = 15), and *S*. *pneumoniae* (72.7%, n = 24). Only, 7.7% of *S*. *aureus* isolates showed inducible clindamycin resistance. Aging (AOR: 3.248, 95% CI: 1.001–10.545, p = 0.050), a history of pneumonia (AOR: 7.004, 95% CI: 3.591–13.658, p = 0.001), alcohol use (AOR: 6.614, 95% CI: 3.399–12.872, p < 0.001), and overcrowded living conditions (AOR: 4.348, 95% CI: 1.964–9.624, p = 0.001) were significantly associated with culture-positive sputum.

**Conclusion and recommendations:**

This study found a high prevalence of bacteria-caused community-acquired pneumonia among adults and low susceptibility to ampicillin, tetracyclines, and amoxicillin-clavulanic acid. Therefore, culture-based bacterial identification and local antibiotic susceptibility testing should be performed regularly. Additionally, new insights into vaccine coverage against highly multi-drug resistant bacteria, particularly *K*. *pneumoniae*, are necessary.

## Introduction

Pneumonia is an inflammation of the lung parenchyma caused by an infectious agent. It is the most common disease with a high prevalence in the community and causes significant morbidity and mortality. The two major categories of pneumonia are community and hospital-acquired [1–3]. The Infectious Diseases Society of America (IDSA) defines community-acquired pneumonia (CAP) as an acute infection of the pulmonary tissue accompanied by the presence of an acute infiltrate on chest radiograph or auscultatory findings consistent with pneumonia in a patient who did not acquire it from a health care system or within the first 48 hours after hospitalization [4].

Globally, CAP is a major cause of morbidity and mortality [5, 6]. The annual incidence of CAP in Europe has been reported to be 1.6–10.6 per 1000 adults [7], and it is estimated to kill nearly one million adults per year in Asia [8]. In Africa, the mortality rate of CAP in adult patients varies between 6% and 15%. Sub-Saharan Africa also shows high levels of morbidity and mortality, with approximately 4 million cases of pneumonia occurring annually, resulting in about 200,000 deaths [9–11]. According to various reports in Ethiopia [12–15], the prevalence of bacterial CAP ranges from 38.7% to 45%.

Infection of CAP transmitted through aspiration, inhalation, and the hematogenous spread of pathogenic microorganisms. Individuals with co-morbid diseases are more likely to contract CAP through aspiration [16–18]. Factors predisposing to CAP include old age, co-morbidities (such as chronic obstructive pulmonary disease (COPD), asthma, bronchiectasis, heart disease, diabetes mellitus, immunosuppressive diseases, and stroke), previous history of pneumonia, immunosuppressive drugs, viral respiratory infections, impaired airway protection, and lifestyle factors such as smoking, alcohol consumption, crowded living conditions, poor dental hygiene, and regular contact with children [19, 20].

Bacterial pneumonia is common, but its etiology varies from place to place [21, 22]. Several studies have reported that the common causes of CAP are *S*. *pneumoniae*, *K*. *pneumoniae*, *P*. *aeruginosa*, *E*. *coli*, *H*. *influenzae*, *S*. *aureus*, *L*. *pneumophila*, *C*. *pneumoniae*, and *M*. *pneumoniae*. Among these, *S*. *pneumoniae* is considered the predominant bacterial pathogen across all age groups and accounts for approximately 30% of pneumonia cases [23, 24]. Studies in Ethiopia also indicate that *S*. *pneumoniae* is the most frequently isolated bacterium, followed by *K*. *pneumoniae* and *S*. *aureus* [12, 13, 25].

Respiratory bacterial pathogens have an impact on public health, affecting healthy individuals and immunocompromised hosts, and causing post-viral infections in the community and hospital settings. The presence of capsules in *S*. *pneumoniae*, *K*. *pneumoniae*, and *H*. *influenzae* confers antiphagocytic properties, enhancing bacterial growth in the airways, which leads pneumonia. Pneumolysin, a well-known virulence factor, also interferes with immune system cells and soluble components [26]. The rise in pathogenic nonencapsulated *S*. *pneumoniae* strains and non-typeable *H*. *influenzae* subtypes has become a worldwide concern [27, 28]. During the pneumonia infection, *K*. *pneumoniae* also employ endotoxins that cause fever, changes in blood pressure, and even shock, as well as fimbriae, outer membrane proteins, and iron acquisition systems for the survival and evasion of host immunity [29].

The global problem of antimicrobial resistance is particularly pressing in developing countries, where infectious disease exposure is high, antibiotic overconsumption, poor quality of antibiotics, and cost constraints that prevent the widespread use of antibiotics, newer and more expensive drugs [30]. Antimicrobial resistance to macrolides and other agents used to treat CAP has become increasingly common in gram-positive and gram-negative bacteria [31]. Although newer antimicrobial agents such as omadacycline, delafloxacin, and lefamulin have recently been approved for CAP, experience with them is too limited to be included as first-line agents in the current adult CAP guidelines [32].

Multi-drug resistance (MDR) has been increased all over the world that is considered a public health threat. Several recent investigations have reported the emergence of multi-drug resistant bacterial pathogens from different origins including humans, birds, cattle, and fish that increase the need for routine application of antimicrobial susceptibility testing to detect the antibiotic of choice as well as the screening of emerging MDR strains [33–41].

In Ethiopia, CAP is treated empirically, and the diagnosis is not supported by confirmation of specific etiologic agent(s). This is due to the lack of culture and AST facilities in most health care facilities, as well as the time and cost required for laboratory procedures. Therefore, routine identification and reporting of bacterial pathogens and their local susceptibility to antibiotics would be valuable for reducing pneumonia complications and mortality in adults. However, there are no documented data regarding this part of the study area. Hence, this study aimed to determine the bacterial profiles, antimicrobial susceptibility patterns, and factors associated with CAP among clinically diagnosed adult patients in Gondar, Northwest Ethiopia.

## Materials and methods

### Study design, period, and setting

This institutional-based cross-sectional study was conducted from April to June 2021 at the University of Gondar Comprehensive Specialized Referral Hospital. The hospital is located in Gondar town, in the Central Gondar Administrative Zone, Amhara National Regional State, and it is about 750 km northwest of Addis Ababa, the capital city of Ethiopia. The hospital provides outpatient and inpatient services for more than five million people in Gondar town and the surrounding area. It has nine outpatient departments, 14 inpatient wards with more than 550 beds, and 100 to 120 emergency patients attending each day.

### Population, sample size, and sampling technique

All adults aged ≥18 years who were clinically diagnosed with typical symptoms of CAP and those who consented to participate and could provide a sputum sample were included. However, patients who were under antibiotic treatment, had a history of hospital admission in the past 14 days, and were admitted to the hospital for 48 hours or more before data collection were excluded from the study. A total of 312 adult patients with clinically diagnosed CAP (CD-CAP) were selected using a systematic random sampling.

### Data collection and laboratory methods

Socio-demographic characteristics, clinical information, and other relevant variables were collected using a face-to-face interview technique after written consent was obtained from the study participants. Sputum specimens were collected from adult patients with CAP using a disposable, leak-proof, sterile, wide-mouthed container with a tight-fitting lid. During the specimen collection, each study participant was instructed to breathe deeply, and then cough deeply and vigorously to provide at least 2 mL of sputum specimen into the container provided. Soon after collection, they were transported to the bacteriology laboratory using an icebox and processed within 30 minutes of collection.

### Bacterial identification

Each sputum specimen was inspected macroscopically for color, volume, viscosity, and odor, followed by microscopic evaluation using Gram staining before culture. For culture, sputum specimens with at least 25 polymorphonuclear leukocytes and less than 10 epithelial cells per low-power field were used [42]. Standard microbiological techniques were used for the isolation and identification of bacteria. A purulent portion of the sputum specimen was inoculated on MacConkey agar (Oxoid Ltd., Basingstoke, UK), mannitol salt agar (Oxoid Ltd., Basingstoke, UK), blood agar (Oxoid Ltd., Basingstoke, UK), and chocolate agar (Oxoid Ltd., Basingstoke, UK) plates using a sterile wire loop. The inoculated MacConkey agar and Mannitol salt agar plates were incubated aerobically at 37°C for 24 hours, whereas the inoculated Blood agar and chocolate agar plates were incubated using a 5% CO_2_ generating candle jar at 37°C for 24 hours. The following day, the plates were examined for bacterial growth. To obtain pure colonies, bacterial colonies of identical size, shape, and color were selected and subcultured in fresh medium. After obtaining pure colonies, further characterization was performed using colony morphology, pigment production, hemolysis pattern, and Gram staining. Bacterial species were identified using a panel of biochemical tests (Oxoid Ltd., Basingstoke, UK), including triple sugar iron agar, indole, Simon’s citrate agar, lysine iron agar, oxidase, urea, and motility for gram-negative bacteria, and gram-positive bacteria were identified based on catalase, coagulase, optochin test, and bile solubility test results. In addition, the X and V factors were used to enhance the growth of *H*. *influenzae* [43].

### Antimicrobial susceptibility testing

A modified Kirby-Bauer disk diffusion technique was used for AST of bacterial isolates according to the Clinical Laboratory Standards Institute (CLSI) 2021 guideline [44]. The suspension of confirmed pure isolates was done by taking 3 to 5 colonies and emulsifying them with 3 to 4 mL of normal saline, and adjusting them to 0.5% McFarland standard [43]. Using a sterile cotton swab, sufficient inoculum was taken and distributed on Muller-Hinton agar plates (Oxoid Ltd., Basingstoke, UK) (supplemented with 5% sheep blood if fastidious) with lawn culture technique. After 3 minutes, a set of standard antimicrobial disks was aseptically placed on the inoculated plates and allowed to stand at room temperature for 15 minutes. All inoculated media were incubated aerobically at 37°C for 24 hours. Zones of inhibition were measured using a ruler. Finally, the results were interpreted as susceptible, intermediate, or resistant. Inducible clindamycin resistance was detected among *S*.*aureus* isolates with a simple disk approximation test, commonly referred to as the D-test [45]. The following routinely used antimicrobial disks of different classes were checked: penicillins (penicillin (10 μg), ampicillin (10 μg), oxacillin (1 μg), and piperacillin (100 μg)), beta-lactamase inhibitor combination (amoxicillin-clavulanic acid (20/10 μg)), cephalosporins (ceftriaxone (30 μg), ceftazidime (30 μg), and cefoxitin (30 μg)), tetracyclines (tetracycline (30 μg) and doxycycline (30 μg)), macrolides (azithromycin (15 μg) and erythromycin (15 μg)), fluoroquinolone (ciprofloxacin (5 μg)), folate pathway antagonist (trimethoprim-sulfamethoxazole (1.25 + 23.75 μg) (co-trimoxazole)), aminoglycoside (gentamicin (10 μg)), lincosamide (clindamycin (2 μg)), and phenicol (chloramphenicol (30 μg)). All antibiotics were obtained from Abtek Biologicals, Ltd., Liverpool, UK [44].

### Data management and quality control

Data were collected using a pre-tested questionnaire. All materials, equipment, and procedures were adequately controlled. Pre-analytical, analytical, and post-analytical stages of quality assurance and standard operating procedures (SOPs) were strictly followed. Staining reagents were checked using expiry date and a known sample smear. The manufacturer’s instructions and bacteriological standard procedures were strictly followed during the culture media preparation. The sterility of prepared culture media was checked by incubating 5% of the batch at 35–37°C overnight and evaluated for possible contamination. Performance of all prepared media was also checked by inoculating international standard strains such as *E*. *coli* (ATCC 25,922), *S*. *aureus* (ATCC25, 923), *H*. *influenzae* (ATCC 49,247), and *S*. *pneumoniae* (ATCC 49,619). The potency of tested antibiotics was monitored using control strains. To standardize the inoculums density of bacterial suspension for the susceptibility test, 0.5 McFarland standard was used [43]. Finally, laboratory tests were analyzed after quality control was performed and the method is ensured to be safe.

### Data analysis and interpretation

Data were checked manually for completeness, clarity and edited for its consistency. After cleaning and coding, the data were entered into EPI data version 4.6 and then exported to Statistical Package for Social Science (SPSS) version 20 (IBM-SPSS Inc., Chicago, IL, USA) for analysis. Tables and figures were used to present the results. Logistic regression models were used to determine the association between dependent and independent variables. Bivariate analysis of factors associated with CAP was done, and those with a P-value < 0.2 were subjected to multivariate analysis. At 95% confidence interval, a P-value ≤ 0.05 was considered statistically significant.

### Ethical consideration

Ethical clearance was obtained from the ethical review committee of the School of Biomedical and Laboratory Sciences, College of Medicine and Health Sciences, University of Gondar (Ref. No. SBMLS/2721; February 24, 2021). Additionally, after explaining the importance, purpose, and procedure of the study, written informed consent was obtained from study participants. Any patient who was not willing to take part in the study had the full right to withdraw. They were informed that all data and specimens obtained from them would be kept confidential using codes instead of any personal identifiers and used only for this study. The results were linked to their respective physicians for appropriate management of the cases.

## Results

### Socio-demographic characteristics and clinical data of study participants

A total of 312 CD-CAP adult patients were enrolled, and about two-thirds of the study participants were males (65.4%, n = 204). One hundred forty-eight (47.4%) were between the ages of 18 and 35, with a mean age of 40.74 (± 16.042 standard deviation (SD)) years. The majority (70.2%, n = 219) were married and more than half of them (51.9%, n = 162) were rural residents (Table 1). Besides, nearly one-third (40.1%, n = 125) of the study participants were alcohol users; 29.5% (n = 92) had a previous history of pneumonia; and 44.5% (n = 139) never washed their mouths and teeth. Alternatively, of all study participants, only 5.5% (n = 17) were asthmatic cases, and 5.1% (n = 16) were cigarette smokers (Table 2).

**Table 1:** Socio-demographic characteristics of study participants.

Characteristics		Frequency (%)
Sex	Male	204 (65.4)
	Female	108 (34.6)
Age	18–35	148 (47.4)
	36–49	68 (21.8)
	50–64	68 (21.8)
	≥65	28 (9.0)
Marriage	Single	77 (24.7)
	Married	219 (70.2)
	Separated	16 (5.1)
Residence	Urban	150 (48.1)
	Rural	162 (51.9)
Education	Unable to read and write	151 (48.4)
	Primary school	56 (18.0)
	High school	46 (14.7)
	Above high school	59 (18.9)
Occupation	Government employed	30 (9.6)
	Private employed	57 (18.3)
	Daily laborer	20 (6.4)
	Farmer	90 (28.9)
	Student	46 (14.7)
	Housewife	69 (22.1)

**Table 2:** Clinical and other relevant data of study participants.

Characteristics		Frequency (%)
Cigarette smoking	Yes	16 (5.1)
	No	296 (94.9)
Alcohol consumption	Yes	125 (40.1)
	No	187 (59.9)
Previous history of pneumonia	Yes	92 (29.5)
	No	220 (70.5)
Overcrowded living condition	Yes	64 (20.5)
	No	248 (79.5)
Contact with children	Never	56 (18.0)
	Sometimes	226 (72.4)
	Always	30 (9.6)
Mouth and teeth hygiene	Never	139 (44.5)
	Sometimes	135 (43.3)
	Always	38 (12.2)
Co-morbidities	COPD	12 (3.9)
	Asthmatic case	17 (5.5)
	Diabetes mellitus	5 (1.6)
	Heart disease	16 (5.1)
	No co-morbidities	262 (83.9)

### The distribution of bacterial isolates among adult CAP patients

**Fig 1:**
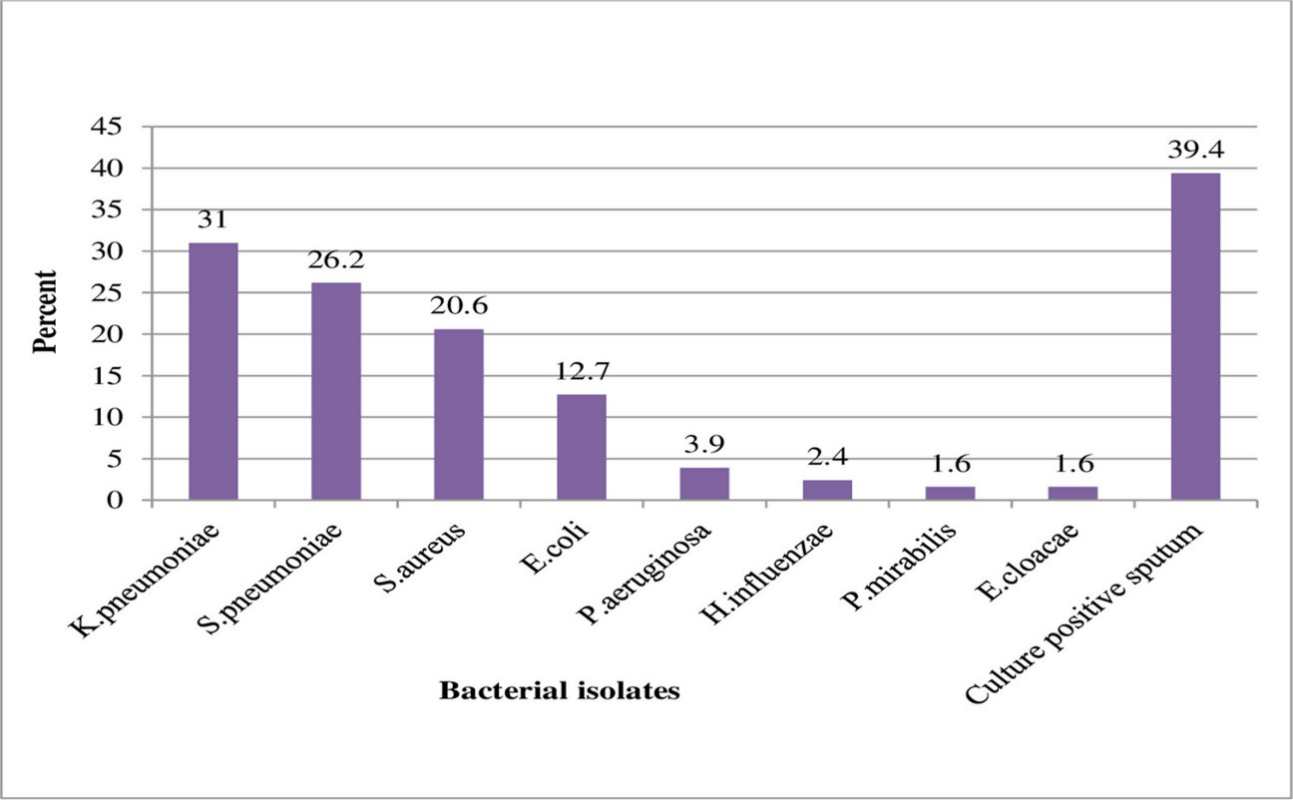
The distribution of bacterial isolates from sputum specimens of CD-CAP adult patients.

In this study, the overall culture-positive sputum for bacterial isolates from clinically diagnosed adult CAP patients was 39.4% (123/312; 95% CI: 34.1%–44.9%). Both gram-positive and gram-negative bacterial isolates were recovered with a 46.8% (n = 59) and a 53.2% (n = 67) prevalence, respectively. Mixed infections were observed among (0.9%, n = 3) patients; *E*. *coli* and *S*. *aureus*, *K*. *pneumoniae* and *H*. *influenzae*, and *K*. *pneumoniae* and *S*. *aureus* were isolated from those three patients. In this study, *K*. *pneumoniae* (31.0%, n = 39) was the most frequently isolated bacteria, followed by *S*. *pneumoniae* (26.2%, n = 33), *S*. *aureus* (20.6%, n = 26), and *E*. *coli* (12.7%, n = 16) (Fig 1).

Phenotypically, isolates were considered *K*. *pneumoniae* when they were pink-red, short, plump, straight rods in Gram staining, as well as large, pink, mucoid, dome-shaped colonies in MacConkey agar plates, and biochemical reactions of lactose-fermenting, hydrogen sulfide negative, gas production, indole negative, citrate positive, lysine positive, oxidase negative, urea positive, and motility negative. Again, *S*. *pneumoniae* isolates were observed as purple, lancet-shaped, spherical bacteria, found in pairs or short chains in Gram stain, and presented as small, grey, moist colonies, alpha-hemolysis on blood agar plates, optochin sensitive, and bile soluble. Furthermore, Gram reaction of *S*. *aureus* isolates revealed purple, spherical, and arranged in clusters resembling grape bunches, as well as mannitol-fermenting, small, golden-yellow colonies were observed in mannitol salt agar.

### Antimicrobial susceptibility patterns of the bacterial isolates

The antimicrobial susceptibility profile of isolates has been presented in Tables 3 and 4. In this study, gram-positive isolates were sensitive to chloramphenicol (100%) and clindamycin (96.6%). *S*. *pneumoniae* isolates were sensitive to clindamycin (97.0%) but resistant to oxacillin (93.9%), tetracycline (87.9%), and doxycycline (84.9%). The antimicrobial susceptibility of *S*. *aureus* isolates to clindamycin, cefoxitin, co-trimoxazole, ciprofloxacin, and erythromycin were 96.2%, 92.3%, 92.3%, 76.9%, and 65.4%, respectively. *S*. *aureus* isolates were penicillin-resistant (80.8%) and 7.7% were methicillin-resistant *S*. *aureus* (MRSA) (Table 3). The recovered gram-negative bacterial isolates showed a high level of susceptibility to some of the tested antimicrobial agents. For instance, 88.7% of them were sensitive to chloramphenicol, followed by gentamicin (87.5%), azithromycin (87.1%), ciprofloxacin (86.6%), and ceftriaxone (79.0%). However, they showed a high level of resistance to ampicillin (100%), tetracycline (87.1%), doxycycline (86.4%), co-trimoxazole (80.6%), and amoxicillin-clavulanic acid (79.0%).

**Table 3:** Antimicrobial susceptibility patterns of gram-positive bacterial isolates.

Antibiotics (%)	Bacterial isolates			
	Pattern	*S*. *pneumoniae* (n = 33)	*S*. *aureus* (n = 26)	Total (n = 59)
CAF	S	33 (100)	26 (100)	59 (100)
	I	0	0	0
	R	0	0	0
CPR	S		20 (76.9)	20 (76.9)
	I	N/A	0	0
	R		6 (23.1)	6 (23.1)
COT	S	16 (48.5)	24 (92.3)	40 (67.8)
	I	0	0	0
	R	17 (51.5)	2 (7.7)	19 (32.2)
TET	S	3 (9.1)	16 (61.5)	19 (32.2)
	I	1 (3.0)	1 (3.9)	2 (3.4)
	R	29 (87.9)	9 (34.6)	38 (64.4)
DOX	S	4 (12.1)	16 (61.5)	20 (33.9)
	I	1 (3.0)	2 (7.7)	3 (5.1)
	R	28 (84.9)	8 (30.8)	36 (61.0)
CLD	S	32 (97.0)	25 (96.2)	57 (96.6)
	I	0	0	0
	R	1 (3.0)	1 (3.8)	2 (3.4)
E	S	15 (45.5)	17 (65.4)	32 (54.2)
	I	0	0	0
	R	18 (54.5)	9 (34.6)	27 (45.8)
OXA	S	2 (6.1)		2 (6.1)
	I	0	N/A	0
	R	31 (93.9)		31 (93.9)
PEN	S		5 (19.2)	5 (19.2)
	I	N/A	0	0
	R		21 (80.8)	21 (80.8)
CXT	S	N/A	24 (92.3)	24 (92.3)
	R		2 (7.7)	2 (7.7)

**Note.** N/A: Not applicable, CPR: Ciprofloxacin, TET: Tetracycline, CAF: Chloramphenicol, E: Erythromycin, DOX: Doxycycline, PEN: Penicillin, COT: Co-trimoxazole, OXA: Oxacillin, CLD: Clindamycin, CXT: Cefoxitin, S: Sensitive, I: Intermediate, R: Resistant.

**Table 4:** Antimicrobial susceptibility patterns of gram-negative bacterial isolates.

Antibiotics (%)	Bacterial isolates							
	Pattern	*K*. *pneumoniae* (n = 39)	*E*. *coli* (n = 16)	*P*. *aeruginosa* (n = 5)	*H*. *influenzae* (n = 3)	*P*. *mirabilis* (n = 2)	*E*. *cloacae* (n = 2)	Total (n = 67)
CAF	S	35 (89.5)	14 (87.5)		3 (100)	1 (50.0)	2 (100)	55 (88.7)
	I	4 (10.5)	0	N/A	0	0	0	4 (6.5)
	R	0	2 (12.5)		0	1 (50.0)	0	3 (4.8)
AZM	S	36 (92.3)	11 (68.8)	N/A	3 (100)	2 (100)	2 (100)	54 (87.1)
	R	3 (7.7)	5(31.2)		0	0	0	8 (12.9)
COT	S	3 (7.7)	5 (31.2)		1 (33.3)	1 (50.0)	2 (100)	12 (19.4)
	I	0	0	N/A	0	0	0	0
	R	36 (92.3)	11 (68.8)		2 (66.7)	1 (50.0)	0	50 (80.6)
TET	S	3 (7.7)	1 (6.25)		1 (33.3)	0	0	5 (8.1)
	I	2 (5.1)	1 (6.25)	N/A	0	0	0	3 (4.8)
	R	34 (87.2)	14 (87.5)		2 (66.7)	2 (100)	2 (100)	54 (87.1)
DOX	S	4 (10.3)	3 (18.8)			0	0	7 (11.9)
	I	1 (2.5)	0	N/A	N/A	0	0	1 (1.7)
	R	34 (87.2)	13 (81.2)			2 (100)	2 (100)	51 (86.4)
GEN	S	37 (94.9)	14 (87.5)	1 (20.0)		2 (100)	2 (100)	56 (87.5)
	I	0	0	0	N/A	0	0	0
	R	2 (5.1)	2 (12.5)	4 (80.0)		0	0	8 (12.5)
AMC	S	2 (5.1)	6 (37.5)	N/A	3 (100)	0	2 (100)	13 (21.0)
	R	37 (94.9)	10 (62.5)		0	2 (100)	0	49 (79.0)
CTR	S	31 (79.5)	12 (75.0)		3 (100)	1 (50.0)	2 (100)	49 (79.0)
	I	0	1 (6.2)	N/A	0	0	0	1 (1.6)
	R	8 (20.5)	3 (18.8)		0	1 (50.0)	0	12 (19.4)
CPR	S	33 (84.6)	14 (87.5)	4 (80.0)	3 (100)	2 (100)	2 (100)	58 (86.6)
	I	0	0	0	0	0	0	0
	R	6 (15.4)	2 (12.5)	1 (20.0)	0	0	0	9 (13.4)
CAZ	S			1 (20.0)				1 (20.0)
	I	N/T	N/T	0	N/A	N/T	N/T	0
	R			4 (80.0)				4 (80.0)
PIP	S			1 (20.0)				1 (20.0)
	I	N/A	N/A	1 (20.0)	N/A	N/A	N/A	1 (20.0)
	R			3 (60.0)				3 (60.0)
AMP	S	0	0	N/A	0	0	0	
	R	39 (100)	16 (100)		3 (100)	2 (100)	2 (100)	62 (100)

**Note.** N/A: Not applicable, N/T: Not tested, CPR: Ciprofloxacin, CTR: Ceftriaxone, TET: Tetracycline, CAF: Chloramphenicol, AZM: Azithromycin, CAZ: Ceftazidime, DOX: Doxycycline, COT: Co-trimoxazole, PIP: Piperacillin, AMP: Ampicillin, GEN: Gentamicin, AMC: Amoxicillin-clavulanic acid, S: Sensitive, I: Intermediate, R: Resistant.

Most of the isolated *K*. *pneumoniae* showed higher resistance to amoxicillin-clavulanic acid, co-trimoxazole, tetracycline, and doxycycline, with resistance rates of 94.9%, 92.3%, 87.2%, and 87.2%, respectively. But *K*. *pneumoniae* isolates were highly sensitive to gentamicin (94.9%), followed by azithromycin (92.3%), chloramphenicol (89.5%), ciprofloxacin (84.6%), and ceftriaxone (79.5%). *E*. *coli* isolates also showed 87.5% sensitivity to chloramphenicol, gentamicin, and ciprofloxacin each, 75.0% to ceftriaxone, and 68.8% to azithromycin. A high level of resistance to tetracycline (87.5%), followed by doxycycline (81.2%), co-trimoxazole (68.8%), and amoxicillin-clavulanic acid (62.5%) were detected in *E*. *coli* isolates. *P*. *aeruginosa* was sensitive to ciprofloxacin (80.0%), but resistant to gentamicin and ceftazidime (80.0% each), and piperacillin (60.0%) (Table 4).

### Multi-drug resistance patterns of bacterial isolates

Overall, 72.2% (n = 91) of bacterial isolates were multi-drug resistant (MDR; resistance to at least one antibiotic from three or more classes). Among the total MDR, (94.9%, n = 37) was *K*. *pneumoniae*, (72.7%, n = 24) was *S*. *pneumoniae*, (26.9%, n = 7) was *S*. *aureus*, (93.8%, n = 15) was *E*. *coli*, (100%, n = 5) was *P*. *aeruginosa*, (100%, n = 2) was *P*. *mirabilis*, and (50.0%, n = 1) was *E*. *cloacae*. Most bacterial isolates were resistant to four classes of antibiotics (31.0%, n = 39) (Table 5).

**Table 5:** Multi-drug resistance patterns of bacterial isolates.

Bacterial isolates	Level of resistance (number (%))								Total MDR isolates ≥ R3
	R0 (%)	R1 (%)	R2 (%)	R3 (%)	R4 (%)	R5 (%)	R6 (%)	R7 (%)	
*K*. *pneumoniae* (n = 39)	_	2 (5.1)	_	1 (2.6)	23 (58.9)	9 (23.1)	3 (7.7)	1 (2.6)	37 (94.9)
*S*. *pneumoniae* (n = 33)	_	4 (12.1)	5 (15.2)	16 (48.5)	7 (21.2)	1 (3.0)	_	_	24 (72.7)
*S*. *aureus* (n = 26)	2 (7.7)	7 (26.9)	10 (38.5)	7 (26.9)	_	_	_	_	7 (26.9)
*E*. *coli* (n = 16)	_	1 (6.3)	_	5 (31.3)	6 (37.5)	1 (6.3)	2 (12.5)	1 (6.3)	15 (93.8)
*P*. *aeruginosa* (n = 5)	_	_	_	3 (60.0)	2 (40.0)	_	_	_	5 (100)
*H*. *influenzae* (n = 3)	_	2 (66.7)	1 (33.3)	_	_	_	_	_	_
*P*. *mirabilis* (n = 2)	_	_	_	_	1 (50.0)	1 (50.0)	_		2 (100)
*E*. *cloacae* (n = 2)	_	_	1 (50.0)	1 (50.0)	_	_	_		1 (50.0)
Total (n = 126)	2 (1.6)	16 (12.7)	17 (13.5)	33 (26.2)	39 (31.0)	12 (9.5)	5 (3.9)	2 (1.6)	91 (72.2)

**Note.** R0: susceptible to all antibiotics, R1 –R7: resistance to 1, 2, 3, 4, 5, 6, and 7 classes of antibiotics, respectively, ≥ R3: resistance to 3 or more classes of antibiotics, MDR: multi-drug resistance.

### Factors associated with culture-positive sputum among CD-CAP patients

In multivariate analysis, the culture positivity of CAP had a significant association with the age group of 65 years and above (AOR: 3.248, 95% CI: 1.001–10.545, p = 0.050), previous history of pneumonia (AOR: 7.004, 95% CI: 3.591–13.658, p = 0.001), overcrowded living condition (AOR: 4.348, 95% CI: 1.964–9.624, p = 0.001), and alcohol use (AOR: 6.614, 95% CI: 3.399–12.872, p < 0.001) (Table 6).

**Table 6:** Bivariate and multivariate analysis of factors associated with culture-positive sputum among CD-CAP adult patients.

Characteristics		Culture		Bivariate Analysis	Multivariate Analysis	
		Positive (%)	Negative (%)	COR (95% CI)	AOR (95% CI)	p-value
Sex	Male	80 (65.0)	124 (65.6)	0.975 (0.605,1.571)		
	Female	43 (35.0)	65 (34.4)	1		
Age	18–35	56 (45.5)	92 (48.7)	1		
	36–49	30 (24.4)	38 (20.1)	1.297 (0.724,2.323)		
	50–64	18 (14.6)	50 (26.4)	0.591 (0.314, 1.114)		
	≥65	19 (15.5)	9 (4.8)	3.468 (1.468,8.195)	**3.248 (1.001,10.545)**	**0.050**
Residence	Urban	53 (43.1)	97 (51.3)	1		
	Rural	70 (56.9)	92 (48.7)	1.393 (0.882,2.199)	0.614 (0.265,1.424)	0.256
Marriage	Unmarried	29 (23.6)	48 (25.4)	0.604 (0.205,1.784)		
	Married	86 (69.9)	133 (70.4)	0.647 (0.234,1.787)		
	Separated	8 (6.5)	8 (4.2)	1		
Education	Unable to read and write	63 (51.2)	88 (46.6)	1.507 (0.799,2.843)		
	Primary school	24 (19.5)	32 (16.9)	1.579 (0.738,3.378)		
	Secondary school	17 (13.8)	29 (15.3)	1.234 (0.549,2.775)		
	College and above	19 (15.4)	40 (21.2)	1		
Occupation	Government employed	6 (4.9)	24 (12.7)	0.325 (0.118,0.895)	**0.200 (0.048,0.831)**	**0.027**
	Private employed	23 (18.7)	34 (18.0)	0.879 (0.432,1.792)		
	Daily laborer	8 (6.5)	12 (6.3)	0.867 (0.315,2.387)		
	Farmer	40 (32.5)	50 (26.5)	1.040 (0.553,1.957)		
	Student	16 (13.0)	30 (15.9)	0.693 (0.321,1.499)		
	Housewife	30 (24.4)	39 (20.6)	1		
Cigarette smoking	Yes	8 (6.5)	8 (4.2)	1.574 (0.575,4.310)		
	No	115 (93.5)	181 (95.8)	1		
Alcohol consumption	Yes	74 (60.2)	51 (27.0)	4.086 (2.521,6.625)	**6.614 (3.399,12.872)**	**< 0.001**
	No	59 (39.8)	138 (73.0)	1		
Previous history of pneumonia	Yes	59 (48.0)	33 (17.5)	4.358 (2.602,7.300)	**7.004 (3.591,13.658)**	**0.001**
	No	64 (52.0)	156 (82.5)	1		
Overcrowded living condition	Yes	38 (30.9)	26 (13.8)	2.803 (1.595,4.924)	**4.348 (1.964,9.624)**	**0.001**
	No	85 (69.1)	163 (86.2)	1		
Contact with children	Never	15 (12.2)	33 (17.5)	1		
	Sometimes	90 (73.2)	140 (74.1)	0.739 (0.302,1.811)		
	Always	18 (14.6)	16 (8.5)	0.715 (0.333,1.538)		
Mouth and teeth hygiene	Never	66 (53.7)	72 (38.1)	2.016 (0.942,4.314)	1.557 (0.517,4.688)	0.431
	Sometimes	45 (36.6)	91 (48.1)	1.048 (0.484,2.269)		
	Always	12 (9.8)	26 (13.8)	1		
Asthmatic case	Yes	6 (4.9)	11 (5.8)	0.830 (0.299,2.305)		
	No	117 (95.1)	178 (94.2)	1		
Heart disease	Yes	7 (5.7)	9 (4.8)	1.207 (0.437,3.330)		
	No	116 (94.3)	180 (95.2)	1		

**Note.** COR: Crude odds ratio, AOR: Adjusted odds ratio, CI: Confidence interval.

## Discussion

Community-acquired bacterial pneumonia is the most frequent cause of pulmonary infection in adults and the increased antimicrobial resistance becomes a serious public health concern [46]. In this study, the overall prevalence of culture-positive sputum of bacterial CAP among adult patients was 39.4% (123/312; 95% CI: 34.1%–44.9%). This finding is comparable with previous Ethiopian studies in Bahir Dar (40.3%) [12], Dessie (38.7%) [14], Arba Minch (40.0%) [15], and findings from Sudan (42.0%) [47]. However, our finding is lower than other findings reported in Jimma, Ethiopia (45.0%) [13], India (48.0%) [48], Tripura, North-eastern India (58.8%) [49], Egypt (50.4%) [16], China (55.1%) [50], and Saudi Arabia (46.6%) [51]. There was some documented evidence in Bangladesh (27.61%) [52] and Tanzania (20.4%) [53], which was lower than our finding. This disparity could be attributed to differences in geographic location, study period, and study population sampling and methods.

Sixty-seven (53.2%) of the isolates were gram-negative bacteria, while 59 (46.8%) were gram-positive bacteria, indicating the predominance of gram-negative bacteria in causing CAP. Comparable findings documented by other studies in Ethiopia [13, 14]. This is due to the presence of various virulence factors dedicated to colonization and invasion of the respiratory airways, as well as prior use of ineffective antibiotics by people outside the hospital, which results in the selection of gram-negative bacteria and increased resistance gene transmission.

In this study, the most frequent etiologic agent isolated from adult patients with CD-CAP was *K*. *pneumoniae* (31.0%). Similarly, *K*. *pneumoniae* was the most commonly isolated bacteria from previous studies in India (29.1%) [48], China (27.4%) [50], Tanzania (29.9%) [53], and Dessie (28.0%) [14]. However, our finding is significantly higher compared to studies reported in Tripura, North-eastern India (20.4%) [49], Bangladesh (13.3%) [52], Saudi Arabia (12.0%) [51], Jimma (11.7%) [13], Arba Minch (4.7%) [15], and Bahir Dar (18.0%) [12]. But it is lower than a study finding in Sudan reported by Ibrahim A [47], which reported a 42.8% prevalence. This is due to their high prevalence in the hospital settings, which can spread to the community through contaminated hands of healthcare personnel, patient visitors, and hospital wastewater, as well as their ubiquitous nature, which can be found in the environment and human mucosa, their capsular nature, the establishment of hypermucoviscous variants, and the acquisition of antibiotic resistance genes. Additionally, a high intake of alcohol in the study participants that promote alcohol-mediated dysbiosis also contributes to *K*. *pneumoniae* infection [54].

*S*. *pneumoniae* was the second most frequently isolated bacteria, accounting for 26.2% of the isolates. This result is comparable with the studies conducted in India (13.3%) [48], Bangladesh (19.1%) [52], Dessie (24.8%) [14], Jimma (28.3%) [13], and Arba Minch (11.8%) [15]. However, our finding is higher than studies in Tripura, North-eastern India (4.2%) [49], and China (9.4%) [50]. Conversely, this finding was lower than the studies in Saudi Arabia (34.0%) [51] and Bahir Dar (35.9%) [12]. Although the presence of vaccines decreased the burden of *S*. *pneumoniae*, the emergence of non-vaccine *S*. *pneumoniae* serotypes due to serotype replacement and/or capsular switching might be the possible reason for their increased percentage in adults.

*S*. *aureus* accounted for 20.6%, which is comparable with other studies [12–14, 47, 49–51] but higher than studies reported in Bangladesh [52] and Arba Minch [15]. In our study, *E*. *coli* accounted for 12.7%. This finding is comparable with the studies conducted in China [50], Tanzania [53], Saudi Arabia [51], Jimma [13], and Bahir Dar [12]. But it is higher than the studies in Tripura, North-eastern India [49], Sudan [47], Bangladesh [52], and Arba Minch [15]. The varying proportion of bacterial isolates among several studies in different areas might be attributed to the variation in the geographic distribution of bacterial isolates, associated risk factors, sample size, type of specimen used, collection and processing of specimens, and the methods used in each investigation.

In this study, the antimicrobial susceptibility patterns of the recovered bacterial isolates were assessed, and the predominant isolate, *K*. *pneumoniae*, was 87.2% resistant to tetracycline and doxycycline. This finding is comparable with local studies conducted in Bahir Dar [12], Arba Minch [25], Jimma [13], and Dessie [14]. High resistance of *K*. *pneumoniae* was observed to the commonly prescribed antibiotic, amoxicillin-clavulanic acid (94.9%). This is consistent with studies conducted in Ethiopia and elsewhere [12, 14, 16, 52]. In the present study, higher resistance by *K*. *pneumoniae* was observed against co-trimoxazole (92.3%). This is comparable with studies conducted in Egypt (89.7%) [16] and Jimma (100%) [13]. However, this finding is in contrast to studies conducted in Dessie (88.8%) [14] and Bahir Dar (90.0%) [12] sensitivity of *K*. *pneumoniae* isolates to co-trimoxazole. Moreover, we also found that most of the isolates were sensitive to ciprofloxacin (84.6%), which is comparable with studies conducted in Ethiopia [12–14, 25]. This variation may be due to the difference in drug-resistant bacterial strains in the local community and the magnitude of the isolated bacteria in studies.

*S*. *pneumoniae* from this study showed 93.9% resistance to oxacillin, which is comparable with a study in Egypt (82.6%) [16] but higher than the studies conducted in Bahir Dar (56.7%) [12], Dessie (56.4%) [14], Jimma (55.0%) [13], and Arba Minch (60.0%) [25]. All the isolates were 100% sensitive to chloramphenicol, which is comparable with studies in Arba Minch (95.0%) [25], Jimma (95.0%) [13], and Bahir Dar (96.7%) [12]. *S*. *pneumoniae* was resistant to erythromycin (54.5%), which is comparable with a study in Arba Minch (50.0%) [25]. This is in contrast to the studies conducted in Bahir Dar [12] and Jimma [13], where *S*. *pneumoniae* was 96.7% and 95.0% sensitive to erythromycin, respectively. In this study, the resistance of *S*. *aureus* to penicillin was 80.8%, which is comparable to studies conducted in Bahir Dar (75.0%) [12], Dessie (75.9%) [14], Arba Minch (83.3%) [25], and Jimma (81.3%) [13]. But this finding is higher than a study in Sudan that found (60.0%) [47]. In this study, 7.7% of *S*. *aureus* was MRSA using cefoxitin as a screening method, supported by other studies [14, 16] in which community-acquired MRSA became an important causative agent of bacterial pneumonia in the study area.

Currently, resistance to the available antibiotics has increased in *K*. *pneumoniae* through changes in cell permeability (due to changes in the KpnEF efflux pump systems and AcrAB-TolC, assuming the loss of porins KpnO), target gene mutation, plasmid-mediated resistance, production of enzymes such as beta-lactamases, carbapenemase, and aminoglycoside-modifying enzymes with various activities (adenylation, acetylation, or phosphorylation) [55]. Changes in penicillin-binding proteins, changes in ribosomal target sites, changes in the bacterial genome, enhanced efflux, and the acquisition of plasmid-encoded genes are the common resistance mechanisms of *S*. *pneumoniae* to beta-lactams, macrolides, fluoroquinolones, and co-trimoxazole [56]. The release of the beta-lactamase enzyme in *S*. *aureus* is the primary cause of penicillin resistance, whereas the *mecA* gene, which codes for the synthesis of penicillin-binding protein, is responsible for methicillin resistance [57].

Interestingly, among the 20.6% (n = 26) of *S*. *aureus* isolates, (7.7%, n = 2) were positive for inducible clindamycin resistance. This indicates that clindamycin therapy for *S*. *aureus* infection results in treatment failure. This could be related to the rapid spread of *erm* genes coding for antibiotic resistance, which facilitates the production of the methylase enzyme, which alters the ribosomal target site in clindamycin-sensitive Staphylococcal strains. Tetracycline resistance was observed in 87.5% of *E*. *coli*, which is comparable to studies in Jimma (100%) [13] and Bahir Dar (90.0%) [12]. In this study, *E*. *coli* showed 75.0% sensitivity to ceftriaxone. This is lower than the studies in Bahir Dar [12], Arba Minch [25], and Jimma [13], which showed 100% sensitivity to ceftriaxone.

The prevalence of MDR was found to be 72.2%, and this is comparable to the studies conducted in Egypt (76.2%) [16], Bahir Dar (76%) [12], Dessie (63.1%) [14], Jimma (62.7%) [13], and Arba Minch (60.3%) [25]. A high level of MDR was observed among *K*. *pneumoniae* (94.9%), followed by *E*. *coli* (93.8%) and *S*. *pneumoniae* (72.7%). Comparable findings were reported in Bahir Dar [12], (*K*. *pneumoniae*, 100%*)*, (*E*. *coli*, 90.0%), and (*S*. *pneumoniae*, 55.0%), Egypt [16], (*K*. *pneumoniae*, 89.7%), (*E*. *coli*, 87.5%), and (*S*. *pneumoniae*, 82.6%), Dessie [14], (*K*. *pneumoniae*, 97.7%*)*, and Arba Minch [25], (*K*. *pneumoniae*, 100% and *E*. *coli*, 100%*)*. The high prevalence of MDR might be related to different factors, such as misuse and overuse of antimicrobials, poor adherence to treatment, poor infection control in health care and community settings, and poor hygiene and sanitation [58].

In our study, risk factors like alcohol use (p < 0.001), previous history of pneumonia (p = 0.001), crowded conditions (p = 0.001), and aging (p = 0.050) were significantly associated with CAP. In different studies, determinants like alcohol consumption [12, 14, 19, 59], crowded conditions [12, 19], aging [14], and previous history of pneumonia [60, 61] were reported as possible risk factors for CAP. The weakening of the immune status of the patient in the older age may be the possible reason. Besides, indoor air pollution due to crowded living or the presence of undernourishment results in high transmission of the disease. Moreover, because alcohol is sedative and suppresses cough and gag reflexes, there is a substantial risk of pathogen aspiration. Alcohol also impairs the phagocytic function of macrophages and neutrophils recruitment, as well as minimizes bacterial clearance in the lungs. Whereas, working at government institutions (p = 0.027) was protective for CAP [62]. This might be due to the lower exposure to risk factors like cigarette smoking, alcohol consumption, and being in a crowded environment.

Although there are strengths, the current study did not attempt to identify some common atypical pathogens that cause CAP like *Chlamydia*, *Mycoplasma*, and *Legionella* species, perform *H*. *influenzae* serotyping, and perform a minimum inhibitory concentration method of antimicrobial susceptibility testing due to resource limitation. Additionally, polymerase chain reaction-based detection of virulence genes and antimicrobial resistance genes were not conducted because our laboratory has no facility for molecular analysis.

## Conclusion and recommendations

In this study, there was a high prevalence (39.4%) of culture-confirmed CAP among adults with frequently isolated pathogens of *K*. *pneumoniae*, *S*. *pneumoniae*, and *S*. *aureus*. The most effective antibiotics against gram-positive bacteria were chloramphenicol and clindamycin, whereas gentamicin, azithromycin, ciprofloxacin, and ceftriaxone were effective against gram-negative bacteria. Gram-negative bacteria were highly ampicillin resistant, followed by tetracyclines, co-trimoxazole, and amoxicillin-clavulanic acid. The predominant isolate, *K*. *pneumoniae*, showed a higher level of MDR (94.9%) and it was resistant to amoxicillin-clavulanic acid, co-trimoxazole, tetracycline, and doxycycline at 94.9%, 92.3%, 87.2%, and 87.2%, respectively. These high figures require rational use of antimicrobial agents and avoidance of self-medication. Therefore, regular culture and local susceptibility testing, and special attention such as vaccine coverage, are essential for better management of CAP in the study area. Culture-positive sputum of adult CD-CAP patients was associated with aging, a history of pneumonia, overcrowded living style, and alcohol use and changing these lifestyle factors would prevent the infection.

## Supporting information

S1 Dataset Dataset used for analysis of the result. (SAV)

S1 Protocol The AST interpretation chart (extracted from CLSI, 2021). (DOCX)

S2 Protocol Laboratory data collection form. (DOCX)

## Abbreviations

AST: Antimicrobial susceptibility testing
ATCC: American type culture collection
CAP: Community-acquired pneumonia
CD-CAP: Clinically diagnosed community-acquired pneumonia
CLSI: Clinical and laboratory standards institute
COPD: Chronic obstructive pulmonary disease
IDSA: Infectious diseases society of America
LRTIs: Lower respiratory tract infections
MDR: Multi-drug resistant/ce
MRSA: Methicillin-resistant *Staphylococcus aureus*
SOPs: Standard operating procedures
SPSS: Statistical package for social sciences
WHO: World health organization
