# Supplementary material for: Bacterial profile, antimicrobial susceptibility patterns, and associated factors of community-acquired pneumonia among adult patients in Gondar, Northwest Ethiopia: A cross-sectional study
Source: PLoS One. 2022 Feb 1;17(2):e0262956. doi: 10.1371/journal.pone.0262956 (PMC8806065; doi:10.1371/journal.pone.0262956)
Supplement: S1 Protocol — (DOCX) [file pone.0262956.s002.docx]

**S2 Protocol. The AST interpretation chart (extracted from CLSI, 2021).**

AST for Enterobacteriaceae

| S. No | Drug Name | Susceptible (mm) | Intermediate (mm) | Resistant  (mm) | Remark |
| --- | --- | --- | --- | --- | --- |
| 1 | Chloramphenicol | 18 | 13-17 | 12 |  |
| 2 | Azithromycin | 13 |  | 12 |  |
| 3 | Cotrimoxazole | 16 | 11-15 | 10 |  |
| 4 | Tetracycline | 15 | 12-14 | 11 |  |
| 5 | Doxycycline | 14 | 11-13 | 10 |  |
| 6 | Gentamicin | 15 | 13-14 | 12 |  |
| 7 | Amoxicillin-clavulanic acid | 25 |  | 18 |  |
| 8 | Ceftriaxone | 23 | 20-22 | 19 |  |
| 9 | Ciprofloxacin | 26 | 22-25 | 21 |  |
| 10 | Ceftazidime | 21 | 18-20 | 17 |  |
| 11 | Ampicillin | 17 |  | 13 |  |

AST for *S. aureus*

| S. No | Drug Name | Susceptible (mm) | Intermediate (mm) | Resistant (mm) | Remark |
| --- | --- | --- | --- | --- | --- |
| 1 | Doxycycline | 16 | 13-15 | 12 |  |
| 2 | Ciprofloxacin | 21 | 16-20 | 15 |  |
| 3 | Chloramphenicol | 18 | 13-17 | 12 |  |
| 4 | Co-trimoxazole | 16 | 11-15 | 10 |  |
| 5 | Penicillin | 29 |  | 28 |  |
| 6 | Tetracycline | 19 | 15-18 | 14 |  |
| 7 | Erythromycin | 23 | 14-22 | 13 |  |
| 8 | Clindamycin | 21 | 15-20 | 14 |  |
| 9 | Cefoxitin | 21 |  |  |  |

AST for *S. pneumoniae*

| S. No | Drug Name | Susceptible  (mm) | Intermediate (mm) | Resistant (mm) | Remark |
| --- | --- | --- | --- | --- | --- |
| 1 | Doxycycline | 28 | 25-27 | 24 |  |
| 2 | Chloramphenicol | 21 |  | 20 |  |
| 3 | Co-trimoxazole | 19 | 16-18 | 15 |  |
| 4 | Tetracycline | 28 | 25-27 | 24 |  |
| 5 | Erythromycin | 21 | 16-20 | 15 |  |
| 6 | Clindamycin | 19 | 16-18 | 15 |  |
| 7 | Oxacillin | 20 |  |  |  |

AST for *P. aeruginosa*

| S. No | Drug Name | Susceptible (mm) | Intermediate (mm) | Resistant (mm) | Remark |
| --- | --- | --- | --- | --- | --- |
| 6 | Gentamicin | 15 | 13-14 | 12 |  |
| 7 | Piperacillin | 21 | 15-20 | 14 |  |
| 9 | Ciprofloxacin | 25 | 19-24 | 18 |  |
| 10 | Ceftazidime | 18 | 15-17 | 14 |  |

AST for *H. influenzae*

| S. No | Drug Name | Susceptible (mm) | Intermediate (mm) | Resistant (mm) | Remark |
| --- | --- | --- | --- | --- | --- |
| 1 | Ampicillin | 22 | 19-21 | 18 |  |
| 2 | Chloramphenicol | 29 | 26-18 | 25 |  |
| 3 | Co-trimoxazole | 16 | 11-15 | 10 |  |
| 4 | Tetracycline | 29 | 26-28 | 25 |  |
| 5 | Augmentin | 20 |  | 19 |  |
| 6 | Ceftriaxone | 26 |  |  |  |
| 7 | Ciprofloxacin | 21 |  |  |  |
| 8 | Azithromycin | 12 |  |  |  |
