# Supplementary material for: Bacterial profile, antimicrobial susceptibility patterns, and associated factors of community-acquired pneumonia among adult patients in Gondar, Northwest Ethiopia: A cross-sectional study
Source: PLoS One. 2022 Feb 1;17(2):e0262956. doi: 10.1371/journal.pone.0262956 (PMC8806065; doi:10.1371/journal.pone.0262956)
Supplement: S2 Protocol — (DOCX) [file pone.0262956.s003.docx]

**S3 Protocol. Laboratory data collection form**

| Code number | | |  | | | | | | |
| --- | --- | --- | --- | --- | --- | --- | --- | --- | --- |
| Hospital card number | | |  | | | | | | |
| Date of specimen collection | | |  | | | | | | |
| Health Organization | | |  | | | | | | |
| S. No |  | | Code | | | | | | Response |
| 1. | Bacterial isolate | | 1*=S. pneumoniae 2*= *S. aureus 3=K. pneumoniae* 4=*E. coli* 5=*P. aeruginosa 6*=*H. influenzae*  7=*P. mirabilis* 8= *E. cloacae* | | | | | |  |
| 3. Antimicrobial susceptibility pattern | | | | | | | | | |
| Antibiotics | | AST results (CLSI, 2021): Zone diameter breakpoints nearest to whole millimetre | | | | | | | |
|  |  | Content | | S | I | R | Reading(mm) | Interpretation | |
| Ceftriaxone | | 30µg | |  |  |  |  |  | |
| Ciprofloxacin | | 5µg | |  |  |  |  |  | |
| Tetracycline | | 30µg | |  |  |  |  |  | |
| Chloramphenicol | | 30µg | |  |  |  |  |  | |
| Erythromycin | | 15µg | |  |  |  |  |  | |
| Doxycycline | | 30µg | |  |  |  |  |  | |
| Penicillin | | 10µg | |  |  |  |  |  | |
| Gentamicin | | 10µg | |  |  |  |  |  | |
| Co-trimoxazole | | 1.25+23.75 μg | |  |  |  |  |  | |
| Ampicillin | | 10µg | |  |  |  |  |  | |
| Clindamycin | | 2 µg | |  |  |  |  |  | |
| Azithromycin | | 15 µg | |  |  |  |  |  | |
| Augmentin | | 20/10 µg | |  |  |  |  |  | |
| Cefoxitin | | 30µg | |  |  |  |  |  | |
| Piperacillin | | 100 µg | |  |  |  |  |  | |
| Ceftazidime | | 30 µg | |  |  |  |  |  | |
